# Long-term trends of publications in journal of neuro-oncology: a bibliographic study of a core journal in the field of neuro-oncology

**DOI:** 10.1007/s11060-024-04869-y

**Published:** 2024-10-30

**Authors:** S. Farzad Maroufi, S. Parmis Maroufi, Mohammad Sadegh Fallahi, Jason P. Sheehan

**Affiliations:** 1 Neurosurgical Research Network (NRN), Universal Scientific Education and Research Network (USERN), Tehran University of Medical Sciences, Tehran, Iran; 2 Department of Neurosurgery, Tehran University of Medical Sciences, Tehran, Iran; 3 Department of Neurological Surgery, University of Virginia, Charlottesville, USA

**Keywords:** Journal of neuro-oncology, Publication trend, Country-based representativeness, Gender equity

## Abstract

**Purpose:**

The Journal of Neuro-Oncology (JNO), established in 1983, plays a key role in publishing research on brain and spinal cord tumors. This study examines JNO’s publication trends, focusing on country and gender representation to highlight its global impact.

**Methods:**

Statistical analyses were conducted using R. Gender of the first authors was predicted using a gender-guesser, and author affiliations were used to determine publication countries. We introduced a novel Country-Related Diversity (CRD) index to assess the JNO’s representativeness, comparing a country’s JNO publications to its overall neurosurgical output. An index value of 1 indicates proportional representation.

**Results:**

The JNO corpus, spanning from 1983 to 2024, comprises 8,154 documents with an average document age of 14.4 years. The average citation count per document is 28.71, with a rate of 2.16 citations per document per year. JNO’s scientific output has grown significantly, peaking at 397 articles in 2011, with a long-term annual growth rate of 3.7%. The keyword analysis highlights “glioblastoma” as the most frequent term, reflecting the journal’s neuro-oncological focus. Geographically, the U.S. led with 2,535 articles (40.1%), followed by China and Germany. International collaboration rose steadily, with multi-country publications increasing from 4.76% in 1983 to 20.98% in 2024. Analyzing contributions from different countries showed a converging CRD index toward 1 (*P* < 0.01), with U.S. and non-U.S. countries trending similarly. Upper-middle-income countries displayed fluctuating CRD patterns, whereas lower-middle-income countries lagged behind. Authorship analysis showed an increasing trend in co-authorship (*P* < 0.01), with the average number of authors per paper reaching 10.4 by 2024. Gender representation revealed a growing number of female first and senior authors, although males still dominate. By 2024, 32.9% of first authors and 21.6% of senior authors were female, signaling a gradual trend toward gender parity (*P* < 0.01).

**Conclusions:**

The CRD index offers a standardized measure of country-specific research representation in the JNO. The convergence towards 1 reflects balanced international representation. JNO publication also reflects a trend toward gender equity, with a notable rise in female first authors, enhancing global research inclusivity.

## Introduction

Despite extensive research and advancements in therapeutic strategies, central nervous system (CNS) cancers remain a significant global health issue. The worldwide incidence rate of these tumors ranges from 2.1 to 5.8 per 100,000 people annually [1, 2]. CNS tumors are the second leading cause of death in children and the third leading cause of death in adults [3]. As a result of the significant burden posed by CNS tumors, the field of neuro-oncology has emerged as a critical area of medical research and has seen considerable advancements in recent decades [4, 5]. These advancements have been driven by various factors, including the widespread use of molecular tumor profiling, epigenomics, and transcriptomics-based tools, the development of cerebral organoids, and improvements in neuroimaging through the application of radiomics and artificial intelligence [6]. To keep pace with these developments, researchers and clinicians rely on scholarly journals to disseminate new findings, share best practices, and foster collaboration. The *Journal of Neuro-Oncology* (JNO) has become a pivotal platform for sharing research findings, clinical trials, and reviews that shape the understanding and management of CNS tumors.

Bibliographic studies offer a unique lens through which the evolution of academic literature within a specific field can be examined. They provide an overview of trends in publication outputs, the impact of individual articles, citation patterns, and the growth of scholarly collaboration over time. By focusing on bibliographic data, one can uncover the shifting themes, the prominence of certain research areas, and the geographic distribution of contributions [7]. Understanding these trends is essential for researchers, clinicians, and policymakers who rely on scientific literature to guide decision-making and identify emerging areas of importance.

Neuro-oncology publications worldwide have increased significantly over the past three decades. However, there may be disparities in publication trends based on gender and country of origin, as seen in other fields. For instance, a previous study found that the United States leads in neurosurgery publications, followed by Japan, Germany, and the United Kingdom [8]. This higher rate of publications in high-income countries may be due to differences in resource allocation. Additionally, certain journals might exhibit bias by publishing more studies from specific countries, potentially due to variations in peer-review processes or the nature of the submitted articles [9, 10]. Similar trends have been observed in related journals. For instance, a study analyzing publication trends in the *Clinical Neurophysiology* journal found an increase in the number of authors per publication over time, with North American and European countries contributing relatively more to the journal. Furthermore, a positive correlation between GDP and publication rates was observed [11]. As a result, ensuring country-based diversity and representativeness in global research efforts is essential for core journals across various disciplines. In addition, gender disparities have long been evident in scientific literature, particularly in medical fields, which have historically been male-dominated, with fewer women pursuing academic research careers [12]. In recent years, however, there has been a significant shift towards increased gender diversity in research. This change can be attributed to expanded opportunities for women, evolving social norms, and concerted efforts to promote gender equality [13]. A previous study of oncology journals revealed a notable increase in female authorship, rising from 25.5% in 2002 to 31.7% in 2018 [14]. Although women now represent a larger proportion of medical researchers, gender disparities may still persist. It is important for journals to be evaluated for gender diversity in their published articles.

JNO is a peer-reviewed, multidisciplinary journal that comprehensively covers both basic and clinical research on cancers of the CNS. Established in 1983 in the U.S., the journal focuses specifically on brain and spinal cord tumors, serving as a platform for scientists and clinicians to share their findings and insights in the field of neuro-oncology. In this study, we aimed to analyze the publication trends of JNO, with a focus on changes in the countries of publication and the gender of authors. The results of this study will provide valuable insight into the journal’s representativeness and its key role among neuro-oncology and related field journals.

## Methods

### Data acquisition and processing

In June 2024, a search of the Scopus database was conducted to identify all publications in the JNO using the source title, with no limitations on the publication date. Scopus was chosen for journal assessment due to its extensive coverage and the analytical tools it provides. The results retrieved for JNO in Scopus were validated through a similar search in the Web of Science database. Additionally, a search string including terms and synonyms for “Neurosurgery,” “Skull Base,” “Neuro-oncology,” and “Neurointerventional Surgery” was developed to identify relevant neurosurgical journals indexed in Scopus in the time frame of JNO’s publication. After the initial search, the indexed journals were manually reviewed for comprehensiveness, and extraneous publications were excluded.

### Statistical analysis

Statistical analyses were conducted using R software (version 4.4.0, The R Foundation, Austria) and the Bibliometrix R-package [15]. First author names were extracted and analyzed for gender using the gender-guesser 0.2.0 Python package, which has been validated with precision, recall, and accuracy rates exceeding 80% [16–18]. Names with ambiguous gender classifications were excluded from the final list to prevent misclassification. The affiliations of corresponding authors were used to identify the publication countries, and publication trends for the top 20 countries were visualized.

A novel index was introduced to evaluate the country-based representativeness and diversity (CRD) of the JNO. This index adjusts for differences in neurosurgical publication rates between countries, using the following formula:

$$\:CRD=\frac{\frac{Country\:X\:publications\:in\:the\:JNO\:in\:Year\:Y}{Total\:publications\:in\:the\:JNO\:in\:Year\:Y}}{\frac{All\:Neurosurgical\:publications\:of\:Country\:X\:in\:Year\:Y}{All\:Neurosurgical\:publications\:of\:Country\:in\:Year\:Y}}$$

This formula normalizes the proportion of a country’s publications in JNO by comparing it to its share of total neurosurgical publications across all journals. The rationale behind this index is that relying solely on the raw number or proportion of JNO publications from a country could be misleading, as it may not account for the country’s overall research productivity. For example, if a country’s total research output is low in a given year, it may naturally have fewer publications in JNO, which may not accurately reflect underrepresentation. The CRD index provides a relative measure by adjusting for each country’s total neurosurgical output, ensuring that the assessment of representation is contextually fair. An index value of 1 indicates that a country is proportionally represented in the JNO, while values above or below 1 suggest overrepresentation or underrepresentation, respectively, compared to the overall neurosurgical literature. To evaluate the significance of the trends, linear regression analysis and the Mann-Kendall Trend test were applied.

## Results

### Main information

The JNO publication corpus spans from 1983 to 2024, encompassing 8,154 documents with a mean document age of 14.4 years. The majority of these documents (6,635) were classified as journal articles. Additionally, there were 813 review articles, 95 conference papers, 46 editorials, and 287 letters. The average citation count per document was 28.71, equating to an average of 2.16 citations per document per year.

**Fig. 1:**
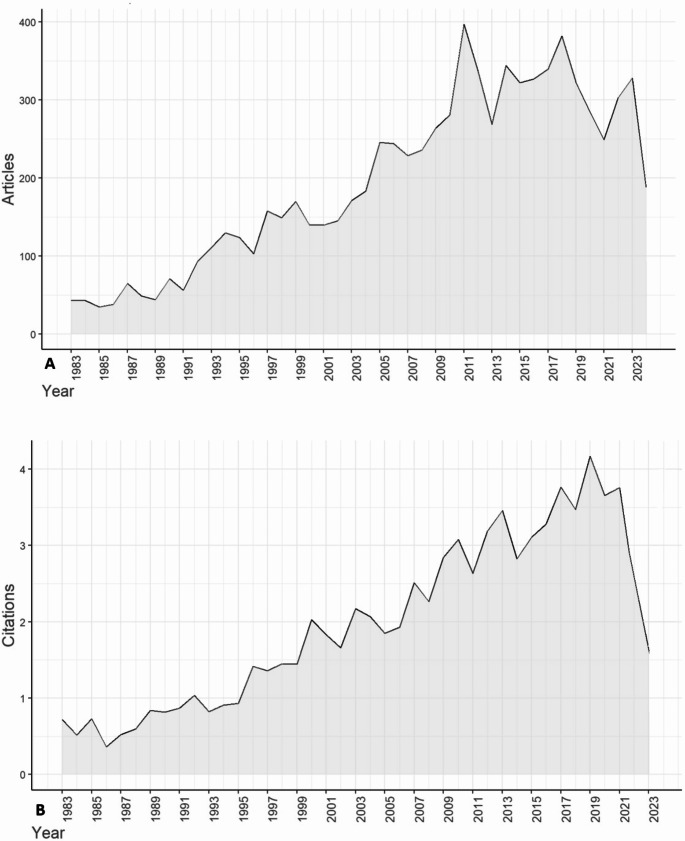
(**A**) Annual scientific production, (**B**) Average annual citation per article in the JNO from 1983 to 2023

Annual scientific output exhibited a significant upward trend over time, rising from 43 articles in 1983 to a peak of 397 in 2011 (Fig. 1. A). Following this peak, production fluctuated around 300 articles per year. The annual growth rate of publications across the journal’s lifespan was 3.7%.

The average article citation per year showed a steady rise (Fig. 1. B). The most cited manuscript, published in 2011 by Maier-Hauff K [19], with a total of 1,106 citations and a rate of 79.0 citations per year. Other highly cited works included publications by Akers JC in 2013 (1,016 citations) and Wiemels J in 2010 (824 citations) [20, 21].

A keyword analysis identified “glioblastoma” as the most frequent Author Keyword, appearing in 1,130 articles. Other prevalent keywords included “glioma,” “chemotherapy,” and “brain tumor,” reflecting the journal’s focus on key areas of neuro-oncological research.

### Publishing countries

**Fig. 2:**
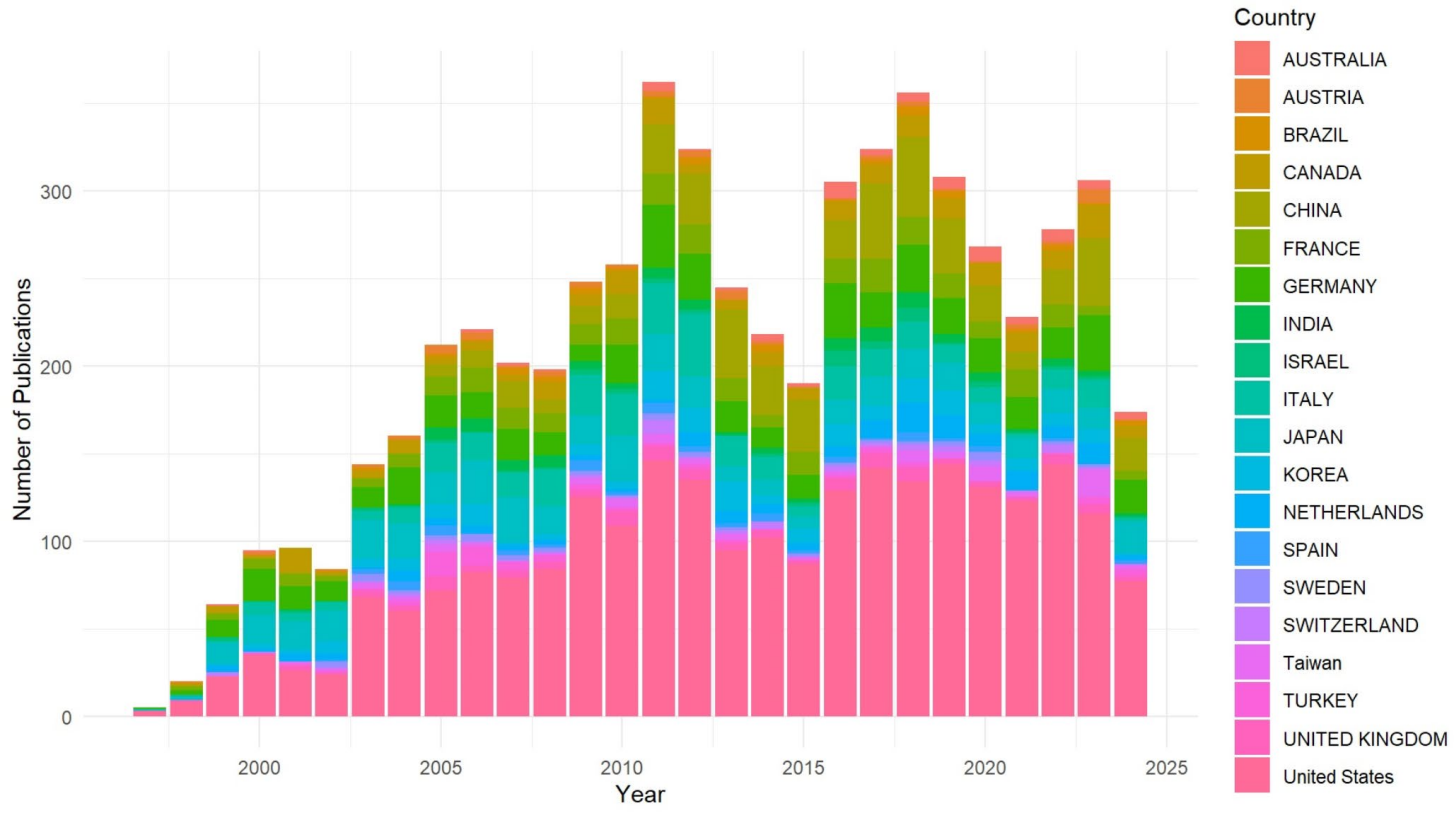
Trend of publication for the top ten countries from 1983 to 2023

The United States was the leading contributor, publishing 2,535 articles (40.1% of the total), followed by China with 548 articles and Germany with 497 articles (Fig. 2). The U.S. also led in total citations, accumulating 80,501 citations, which averaged 31.76 citations per article.

**Fig. 3:**
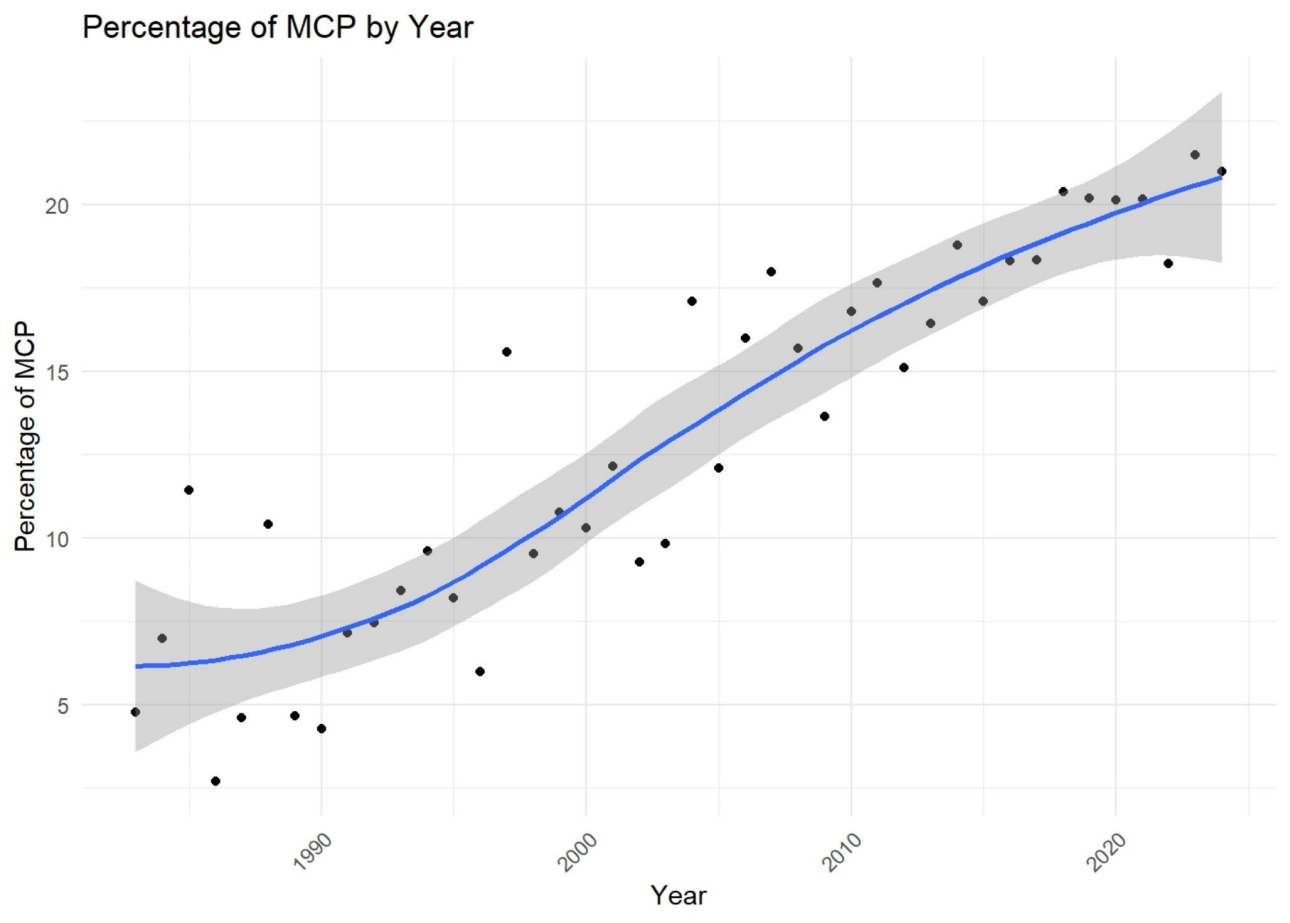
Trend of Multiple Country Publications (MCP) in JNO from 1983 to 2023

The trend in multi-country publications in the JNO indicated a significant rise in international collaboration over the analyzed period. In the early years, the percentage of multi-country publications was relatively low, starting at 4.76% in 1983 and fluctuating between 2.70% and 11.43% throughout the 1980s (Fig. 3). However, from the mid-1990s onward, a notable upward trend emerged, with the percentage of multi-country publications reaching 15.58% in 1997. This growth continued through the 2000s, peaking at 17.98% in 2007, and further increased in recent years, reaching 21.47% in 2023. By 2024, the percentage remained high at 20.98%.

**Fig. 4:**
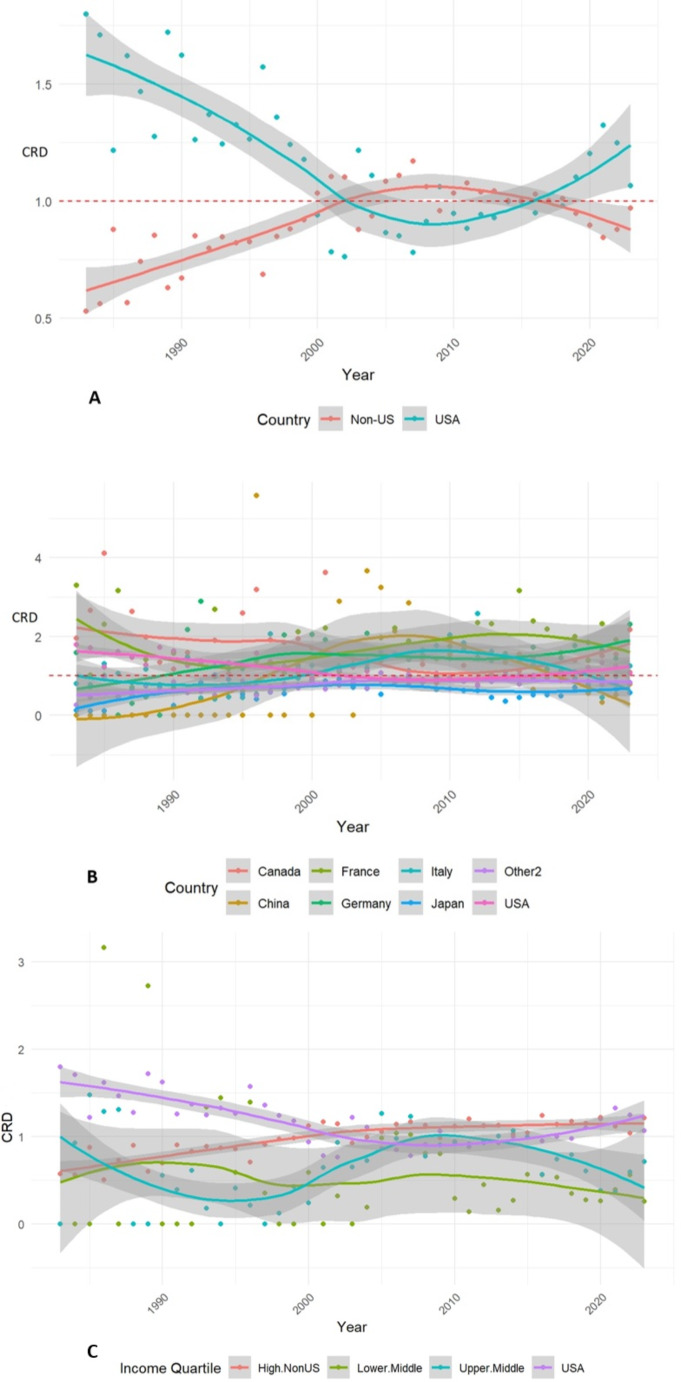
Trend of CRD index change from 1983 to 2023 for (**A**) U.S. and non-U.S. countries, (**B**) for the top 7 countries, (**C**) for countries divided based on world bank income

The number of publications from non-U.S. countries steadily increased throughout the analyzed period (Fig. 2). This growth is particularly notable in countries such as China, Germany, and Japan, which made significant contributions to neuro-oncology research. Although the U.S. consistently held the leading position in terms of JNO publications, the CRD index for both U.S. and non-U.S. countries has been converging toward 1 (*P* < 0.01, Fig. 4. A). An analysis of the top seven countries in total publications compared to the rest of the global JNO output revealed similar trends, with multiple countries approaching a CRD index of 1 and fluctuating around this value (Fig. 4. B). While Canada and the U.S. showed declining trends, other countries demonstrated an upward trajectory.

An additional analysis was conducted based on World Bank income quartiles. Given the large number of publications from the U.S. and its status as the JNO’s publishing country, the U.S. was included as a separate entity. Low-income countries were excluded from the analysis because only one low-income country, Burkina Faso, was represented in the JNO publication sample, with a single paper. While the CRD index for both the U.S. and non-U.S. high-income countries converged, they settled at a slightly higher-than-optimal CRD index of 1.10–1.20 (Fig. 4. C). In contrast, lower-middle-income countries never reached a CRD index of 1 and did not show an upward trend. However, upper-middle-income countries displayed more complex patterns, reaching a CRD index of 1 in both 1980 and 2010.

### Authorship

A total of 29,174 authors contributed to the publications, resulting in 58,956 author appearances. Single-authored documents were rare, with only 297 instances, while the rest involved multiple co-authors.

**Fig. 5:**
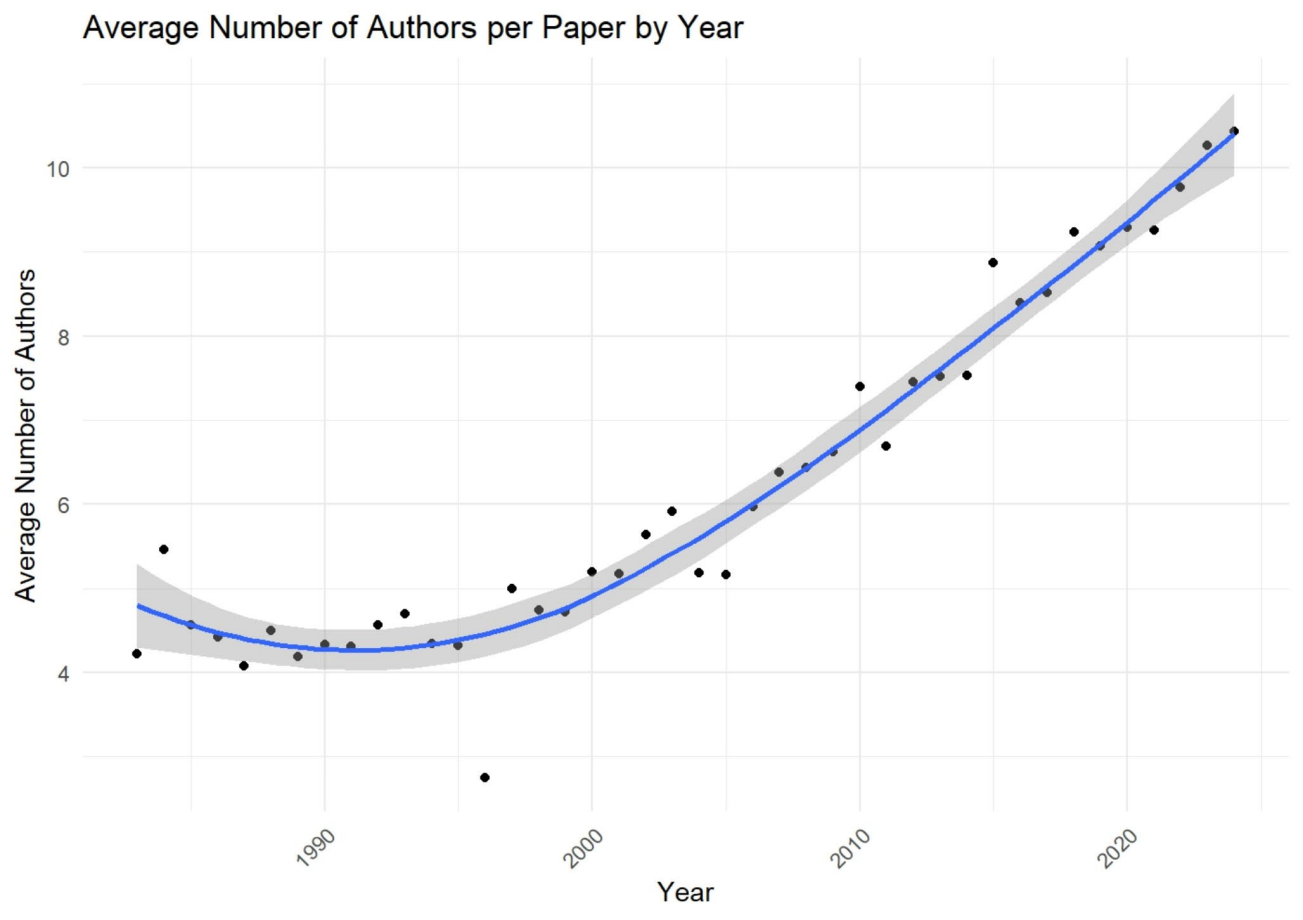
Trend of average number of authors per article from 1983 to 2023

On average, each author contributed to 0.28 documents, and each publication included an average of 7.23 co-authors. The number of authors per article steadily increased over the analyzed period (*P* < 0.01, Fig. 5). In the early years, the average number of authors remained relatively stable, ranging from 4.07 in 1987 to 5.2 in 2000. However, beginning in 2005, a marked increase was observed, with the average exceeding 6 authors per paper by 2007. This upward trend continued, reaching 9.77 authors per article in 2022 and peaking at 10.4 in 2024.

**Fig. 6:**
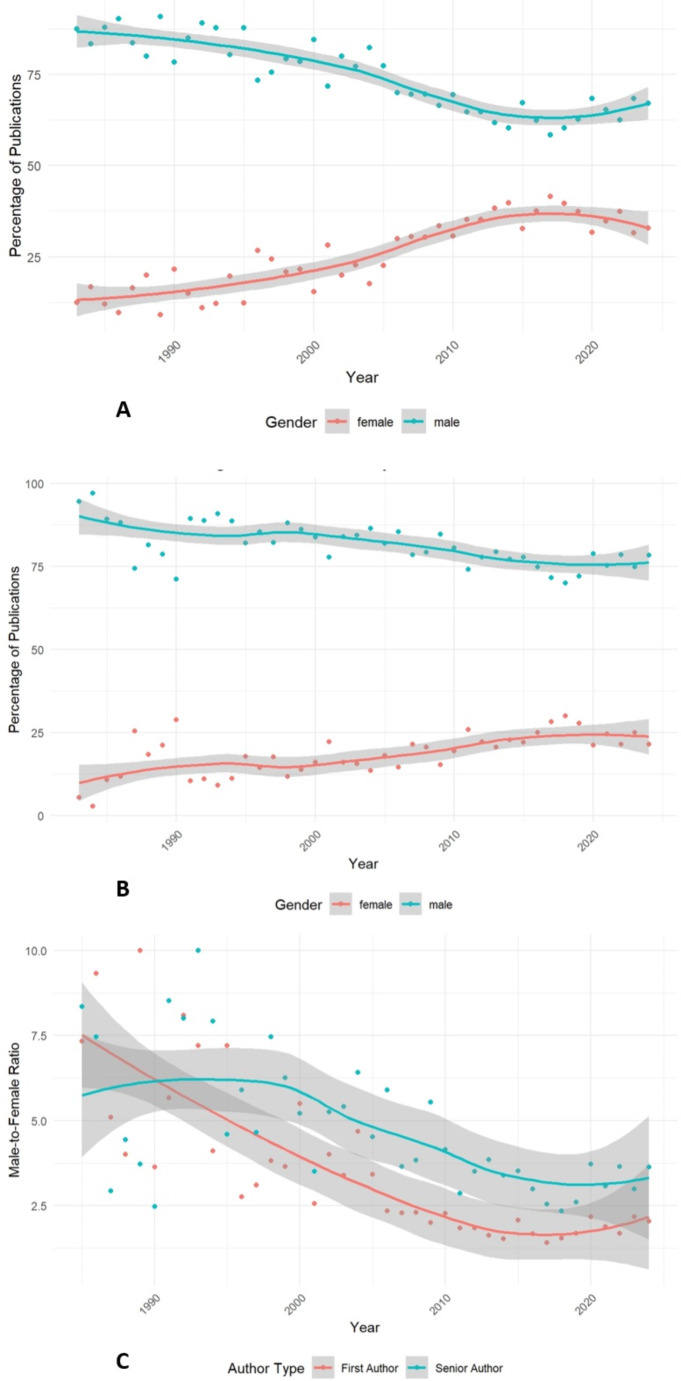
Trend of percentage of male and female (**A**) first-authors and (**B**) senior authors along with the trend of (**C**) male to female ratio for both first and senior authors from 1983 to 2023

The gender distribution of authors in JNO revealed that males constituted the majority of first authors throughout the analyzed period. However, there has been a gradual shift toward a more balanced distribution (Fig. 6. A), with a significant increase in the number of female first authors (*P* < 0.01). Since 2007, the male-to-female ratio among first authors has fluctuated between 1.40 and 2.29. The highest proportion of female first authors was observed in 2017, accounting for 41.6% of annual publications. As of 2024, female authors comprise 32.9%.

Similarly, the gender distribution of senior authors in JNO consistently showed a male majority, though a gradual shift toward gender parity was also noted (Fig. 6. B). The number of female senior authors has steadily increased (*P* < 0.01), with the male-to-female ratio ranging from 2.86 to 3.85 since 2011. The highest percentage of female senior authors was recorded in 2018, at 30.0%. In 2024, female senior authors represent 21.6% of the total. Interestingly, the male-to-female ratio differed significantly between first and senior authors, despite both groups showing a similar rate of change over the study period (Fig. 6. C, *P* < 0.01).

## Discussion

The JNO was established to address the growing need for specialized journals that cover the unique challenges of diagnosing and treating tumors in the central nervous system. Its focus encompasses a wide range of topics including gliomas, meningiomas, brain metastases, treatment modalities such as chemotherapy and radiotherapy, and emerging therapies like immunotherapy. The journal’s impact factor has steadily increased, reflecting its growing influence within the neuro-oncology community. The publication trends in the JNO, as with many academic journals, have evolved over time, reflecting changes in research focus, authorship, and advancements in the field. A systematic review of neuro-oncology authorship trends since 1944, as reported in a 2023 study, has shown a progressive increase in the number of neuro-oncology articles published each year. This increase is more pronounced in medically oriented journals compared to surgically oriented ones [22]. JNO has followed this trend of increased publication volume over time as demonstrated by our findings.

Recent studies have focused on gender representation in authorship. Historically, male authors have dominated both first and senior author positions. However, there has been a trend toward more equitable gender distribution in neuro-oncology literature over time. Despite this progress, female authors still have lower average publication numbers and are less likely to hold first or senior authorship positions compared to their male counterparts [23, 24]. Our study of JNO found similar trends in female authorship, with female first author rates increasing from 12.5% in 1983 to 37.06% in 2024 and senior author rates increasing from 5.41% in 1983 to 21.6% in 2024. Consequently, female authors’ overall contribution has remained lower than that of male authors. Numerous social, cultural, and economic factors have been identified that influence female authorship in academic publications. For instance, women are generally underrepresented in hiring, earnings, funding, leadership positions, and recognition by award committees [25–27]. Additionally, women are more likely to experience authorship disagreements, are less likely to be named on a given article or patent produced by their team, and their works are less likely to be recognized [26, 28]. These factors exacerbate gender inequality in scientific publications. The field of neuro-oncology is not exempt from these effects, and further studies are required to explore the factors contributing to gender disparities in authorship and develop strategies for improvement.

Furthermore, we found that the reduction in the male-to-female ratio among the last authors each year mirrors the ratio observed among the first authors eight years earlier. This timeframe aligns with the typical duration of a neurosurgery residency or a neuro-oncology fellowship following other residencies. The lower rates of female last authors, compared to female first authors, may be due to historical gender disparities in residency and fellowship programs [29], which have been improving in recent years. The effects of developing more inclusive programs in neuro-oncology are evident in our findings. Although the number of women in this field has increased in recent years, the effects on senior authorship positions are still evident. Additionally, a previous study in neurosurgery found that only 12% of neurosurgeons in the US and Canada were female, and while women represent around 12% of neurosurgeons at the assistant and associate professor levels, the rate drops to 5.84% at the full professor level [30]. Similar differences may exist in the field of neuro-oncology, explaining the disparity between female junior and senior authors in JNO.

The number of articles published in leading neurosurgical journals serves as a key indicator of a country’s productivity in neurosurgical and neuro-oncology research. The United States has consistently held a leading position, with a significant presence in top neurosurgical journals [31]. This is in part due to a large number of academic institutions with dedicated neurosurgery departments, substantial research funding, and a high volume of clinical cases contributing to a robust research environment. European nations, particularly those in Western Europe like Germany, the United Kingdom, Italy, and France, are also major contributors to neurosurgical literature. These countries benefit from historic and well-established neurosurgical training programs and strong research networks, facilitating the production of high-quality research. Japan stands out as another significant contributor, boasting a high density of neurosurgeons and a long-standing tradition of research, encompassing both clinical and laboratory settings. Furthermore, several countries with developing economies, including China, India, and Brazil, have demonstrated a recent surge in neurosurgical and neuro-oncology-related research output. This growth likely stems from factors like the expansion of neurosurgical and neuro-oncology programs, increased investments in healthcare infrastructure, and a growing emphasis on research and international collaboration [31–33]. JNO reflects a similar trend, with the United States leading the pack, followed by China, Japan, and Germany. Meanwhile, previous studies have documented regional disparities in medical publications. For example, a 2023 study found that research articles with corresponding authors from the United States, United Kingdom, Canada, or Australia are overrepresented in medical journals. However, this trend is decreasing as publications from other countries, such as China and Israel, are increasing. Additionally, the same study showed a bias towards articles from the journal’s home country. For instance, UK-based journals published a higher proportion of articles from the UK, Australia, Canada, and New Zealand, while American journals favored corresponding authors from the U.S. Authors are also more likely to cite work from their own countries [34]. In this study, we developed a simple and informative index, CRD, to assess a country’s representation in the JNO. CRD is calculated by dividing the percentage of JNO publications from a specific country in a given year by the percentage of all neurosurgery publications from that same country in that year. An index value closer to one indicates that the country is well-represented in both the JNO and broader neurosurgery journals. Our findings demonstrate that as the JNO has gained recognition in the field, evidenced by increasing citations, the journal is also becoming more diverse. This is reflected in a fairer representation of both U.S. and non-U.S. articles.

**Fig. 7:**
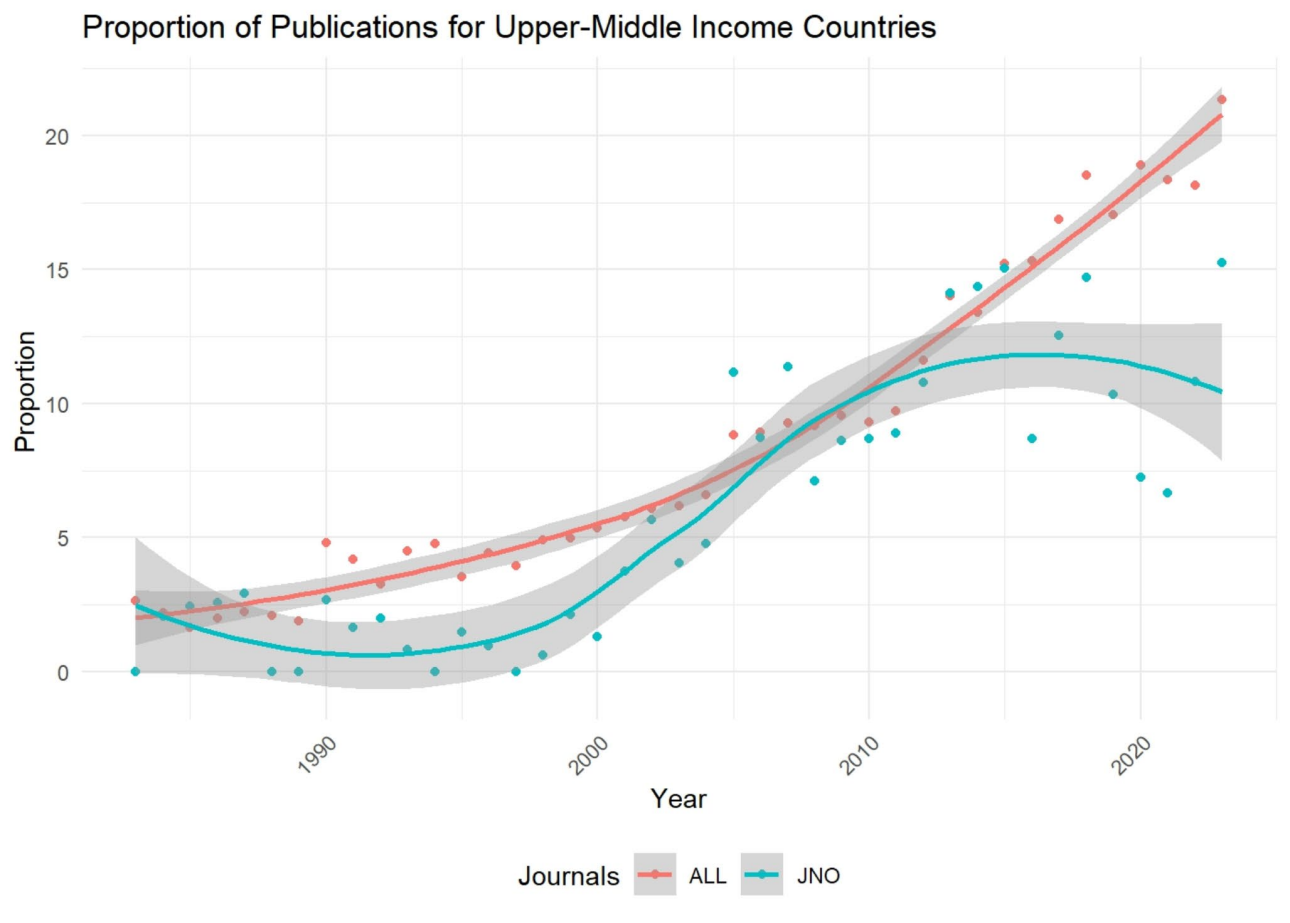
Percentage of JNO and all neurosurgical journals’ publication by upper-middle income countries

We analyzed the geographical distribution of research published in the JNO by dividing countries into four categories: the United States (the journal’s origin), high-income non-US countries, upper-middle-income countries, and lower-middle-income countries. Our findings revealed distinct patterns of representation over time. Initially, the JNO exhibited a disproportionate number of articles from the United States. However, this trend diminished in subsequent years, leading to a fairer representation. Conversely, high-income non-US countries saw a rise in their representation within the JNO, mirroring the growth in their research funding and improved infrastructure for neuro-oncology research. Throughout the analyzed period, lower-middle-income countries remained underrepresented in the JNO. This aligns with a previous study by Young et al. [35], which reported that 94% of neuro-oncology papers from these countries appeared in journals with impact factors below five. This underrepresentation likely stems from limitations in data collection, analysis resources, and trained personnel. Interestingly, upper-middle-income countries displayed a fluctuating pattern of representation. Initially, they had a nearly fair representation which was due to a very limited number of neurosurgical publications from these countries in 1980. Subsequently, the CRD index showed a decrease which corresponds to the rise in the number of publications in the field of neurosurgery while their JNO publications remained nearly constant (Fig. 7). Later with a rising trend, by 2010, their representation reached a fair level as the analysis of publications shows increasing publication in JNO corresponding to increasing research in the field of neuro-oncology. However, recent years have seen a decline in the CRD index, as settling at a plateau is observed in JNO publications while having an increasing number of neurosurgical publications. Several factors might explain these fluctuations in upper-middle-income countries. The JNO’s rising impact and its move to the top quartile of clinical neurology and oncology journals in 2010 (from https://www.scimagojr.com) may have led to stricter acceptance standards which have led to a plateau in the number of JNO publications by upper-middle income countries. Furthermore, advancements in neuro-oncology research often require expensive, high-tech equipment and specialists in more specialized fields. These resources might be limited in upper-middle-income countries, potentially hindering their ability to consistently produce research meeting the JNO’s evolving standards.

In recent years, the fields of neuro-oncology and neurosurgery have witnessed a significant rise in multinational and multicenter studies. Similarly, in JNO, the number of authors per paper and the percentage of multinational publications have increased substantially over time. This increase may be underestimated since some of the authors are trained in countries other than their practicing country. This trend is indicative of a broader move towards global collaboration in medical research and a broader emphasis on larger data studies. Such studies typically involve researchers and institutions from various countries, contributing to a significant pooling of resources, expertise, and patient populations [8, 36]. This collaborative approach offers numerous advantages, including enhanced research quality, greater statistical power, increased generalizability of findings, and the ability to address complex research questions that single-center studies might not be equipped to handle. In the present study, we found the U.S. to be the leading country with the highest rates of multi-national publication in JNO. This is consistent with broader trends in medical research, where the US plays a pivotal role due to its substantial research funding, advanced healthcare infrastructure, and a large number of academic and clinical research centers [31]. Moreover, while the number of publications is a valuable metric, it is not the sole indicator of a country’s impact on neuro-oncology and neurosurgery. Citation rates, research quality, and the development of new therapies and technologies are all equally important factors. The field of neuro-oncology research is constantly evolving. As access to education and research funding improves around the world, we can expect more countries to contribute to the body of neurosurgical knowledge. Furthermore, increasing neuro-oncology and neurosurgical capacity and research in low- and middle-income countries is crucial. This will help address the global disparities in both neuro-oncology care and knowledge.

### Limitations

We acknowledge several limitations of our study. We relied on published articles for analysis. We did not have access to data on submitted papers or ones in the press, which could provide a more comprehensive picture of how different countries are represented. However, the peer review process employed by JNO helps mitigate potential bias through the SNAPP editorial management system, which flags known conflicts of interest. Moreover, our analysis considered the corresponding author’s country affiliation as listed in the published work. This might not always reflect where the research was conducted. We used the “gender-guesser” package to identify the author’s gender based on first names. This method has limitations, including misidentifying non-gender-specific names, conflating sex with gender, and not recognizing non-binary and transgender identities.

While the CRD index offers a clear year-by-year metric for each country’s contribution to JNO, adjusted for the total number of neurosurgical publications and overall neurosurgical output, it has certain limitations that need to be addressed. For countries with a small number of publications, the CRD index may be skewed, leading to outliers where countries with few neurosurgical publications could appear disproportionately over- or underrepresented, despite minimal absolute numbers. This effect is particularly noticeable during the initial years after journal inspection when the overall number of publications is low. For countries with rapidly changing research outputs, the index may not fully capture these fluctuations. For instance, countries experiencing rapid growth in neurosurgical research might show temporary underrepresentation in JNO as their total output increases faster than their representation in the journal, while a sudden decline in research could have the opposite effect. Given these factors, it is important to consider how shifts in a country’s overall research productivity may influence its CRD index over time, particularly for countries with smaller or more variable research programs. Additionally, variations in healthcare policies and infrastructure across countries can lead to some focusing more on clinical neurosurgery, while others concentrate on specialized fields like neuro-oncology, resulting in skewed comparisons. Lastly, it should be noted that the CRD denominator only takes into account the total neurosurgical output of a specific country, while non-neurosurgical authors are also contributing to the JNO hence making deductions regarding the field of neurosurgery in some countries (with more non-neurosurgery affiliated authors) less accurate.

### Future directions

To enhance global representation and gender diversity in the JNO, a combination of proactive strategies and initiatives can be implemented. For instance, diversifying the editorial board to include more members from underrepresented countries, particularly from Africa, Latin America, and Asia, will not only encourage submissions from these regions but also highlight relevant regional challenges in neuro-oncology. Moreover, encouraging papers that address neuro-oncology challenges specific to certain geographic areas (e.g., the prevalence of certain brain tumors in low-resource settings) could ensure that the journal is not Euro-American-centric. Additionally, offering fee waivers or reductions for authors from low-income countries could improve the representation of these countries, as financial barriers can disproportionately affect these groups. Furthermore, establishing a mentorship program that pairs senior researchers with early-career scientists from underrepresented regions and women researchers. This can aid in knowledge transfer, publishing skills, and networking opportunities [37, 38]. Additionally, the journal should publish a Diversity, Equity, and Inclusion (DEI) statement outlining its commitment to diversity and inclusion. This statement can serve as a framework for evaluating progress and accountability in achieving diversity goals [39].

## Conclusion

The JNO has played a significant role in advancing neuro-oncology research since its inception in 1983. It has fostered a collaborative global research environment, drawing contributions from a diverse international community of researchers. The journal has seen a steady increase in publications from non-U.S. countries, reflecting its growing recognition and commitment to inclusivity. The CRD index for both US and non-US countries moving towards 1 signifies a balanced and representative authorship from around the world. Moreover, JNO has consistently promoted diversity in research topics, with “glioblastoma” and other critical neuro-oncological issues being focal points. The journal’s efforts to support gender diversity are also evident, with a notable rise in female first authors over time. Overall, JNO’s comprehensive approach to representativeness and diversity highlights its pivotal role in shaping global neuro-oncology research.

## Data Availability

No datasets were generated or analysed during the current study.
